# The traditional ethnic herb *Tadehagi triquetrum* from China: a review of its phytochemistry and pharmacological activities

**DOI:** 10.1080/13880209.2022.2052908

**Published:** 2022-04-01

**Authors:** Hong-Xia Tang, Wen-Bing Sheng, Xin-Yi Liu, Pei-Wu Cui, Li-Min Gong, Qing-Ling Xie, Wen-Mao Wang, Bin Li, Wei Wang, Xu-Dong Zhou

**Affiliations:** a TCM and Ethnomedicine Innovation & Development International Laboratory, Innovative Materia Medica Research Institute, School of Pharmacy, Hunan University of Chinese Medicine, Changsha, P.R. China; b Meicha Technology Research Center, Hunan Qiankun Biotechnology Co., Ltd., Zhangjiajie, Hunan, P.R. China

**Keywords:** Fabaceae, secondary metabolites, flavonoids, phenylpropanoids, bioactivies

## Abstract

**Context:**

*Tadehagi triquetrum* (Linn.) Ohashi (Fabaceae) (*TT*), is a traditional herbal medicine used especially in China’s ethnic-minority communities, such as the Zhuang, Dai, Li and Wa aeras. As an ethnic medicine, it has long been used to treat various diseases.

**Objective:**

This review summarised the phytochemical and pharmacological progress on *TT* from 1979 to October, 2021 by highlighting its chemical classification, structural features, pharmacological applications and folk applications to provide inspirations and suggestions for accelerating further research of this traditional phytomedicine.

**Methods:**

The information on *TT* in this article has been obtained using these multiple scientific databases including Scifinder, Web of Science, ScienceDirect, Wiley, ACS publications, Springer, PubMed, China Knowledge Resource Integrated Database from the China National Knowledge Infrastructure (CNKI), Google Scholar and Baidu Scholar. Some information was also collected from classic literature on traditional Chinese medicines.

**Results:**

More than 70 compounds have been isolated and reported from *TT* to date by the comprehensive analysis of the current literature. A large number of traditional uses and pharmacological studies have exhibited diversified bioactivities of various *TT* extracts and its metabolites, including anti-inflammatory, antimicrobial, anti-hepatitis B virus, hepatoprotective, insecticidal, etc.

**Conclusions:**

As a famous traditional medicine with a long history, *TT* has various medicinal uses and some of them have been supported by modern pharmacological researches. Further detailed studies on the action mechanisms, pharmacodynamics and structure-function relationships of single compounds or active constituents from *TT* are also required.

## Introduction

The genus *Tadehagi* (Fabaceae), which consists of 6 species in the world, is mainly distributed in tropical Asia, Pacific Islands and Australia. Two of these plants growing only in China, namely *Tadehagi triquetrum* (Linn.) Ohashi and *Tadehagi pseudotriquetrum* (DC.) Ohashi, distributed widely in the southern tropical and subtropical regions of China, such as Guangxi, Guangdong, Hainan, Yunnan, and Hunan province (Wang et al. 2007). *Tadehagi triquetrum* is called ‘Hu-Lu-Cha’ and it is locally known as ‘Tian-Dao-Bing’ (Guangxi province), ‘Bai-Lao-She’ (Guangdong province), ‘Niu-Chong-Cao’ (Hainan province), ‘Lan-Gou-She’ (Jiangxi province), ‘Cha-Bao’ (Zhuang name) in Chinese. So far, according to ‘The Plant List’, *T*. *triquetrum* has 7 accepted synonyms (Table 1). It is a deciduous subshrub and grows on sunny barren slopes, undergrowth, and hilly areas and in thickets along the road with an altitude of 500-1400 m (Editorial Board of Chinese Flora, Chinese Academy of Sciences 1997). It grows 1-2 m tall and has erect stems with triangular prisms and sparsely short bristles. Its flowering period is from July to August and the fruiting period ranges from October to November. In addition, the microstructural characteristics of *TT* are mainly shown in the cross sections of the stems and leaves. (Jiangsu College of New Medicine 2000).

**Table 1.** Synonyms of *T. triquetrum* according to The Plant List (http://www.theplantlist.org).

No.	Synonyms
1	*Desmodium triquetrum* (L.) DC.
2	*Desmodium triquetrum* subsp. *genuinum* Prain
3	*Desmodium triquetrum* subsp. *triquetrum*
4	*Hedysarum triquetrum* L.
5	*Meibomia triquetra* (L.) Kuntze
6	*Pteroloma triquetrum* (L.) Benth.
7	*Tadehagi triquetrum* subsp. *triquetrum*

*TT* has been extensively used in traditional medicine. It was recorded for the first time in ‘Sheng Cao Yao Xing Bei Yao’ and the whole herb of *TT* has been included in the 1977 Edition of the Chinese Pharmacopoeia. According to the theory of traditional Chinese medicine (TCM), it has bitter taste and the property of cool, which possesses the effects of clearing heat, detoxifying, draining dampness, eliminating phlegm, expelling parasites and dispersing blood stasis. It has long been used to treat colds, acute tonsillitis, acute pharyngitis, hookworm disease, lung abscess, nephritis edoema, dysentery, jaundice, rheumatic arthritis and scabies in the form of a single herb or herbal preparation formula (Chen et al. 2016). In particular, people in the Zhuang minority areas of Guangxi province have long used it to treat hepatitis (Peng et al. 2008). In addition, *TT* is considered an edible herb as the famous non-camellia tea, called ‘Hu-Lu-Cha’, especially in Guangdong and Guangxi regions of China (Chen and Mei 2013). Its safety, as the dietary herbal medicine, makes it more common in people's daily life, with the enhancement of health awareness. These above therapic effects have been evident in several official ethnic pharmacography, folk applications and public praise. This ‘evidence-based’ therapeutic history and effects are reflected in the extensive traditional clinical application of *TT* or various *TT* formulations that are commonly sold as over-the-counter drugs or dietary supplements.

Previous phytochemical research on *TT* has revealed the presence of more than 70 secondary metabolites, including flavonoids, phenylpropanoids, phenolic compounds, triterpenoids, steroids and others. Modern pharmacological studies have exhibited diversified pharmacological activities of various *TT* extracts and these metabolites, such as anti-inflammatory, antimicrobial, anti-hepatitis B virus, hepatoprotective, insecticidal, etc. Given the above, *TT* has attracted more attention in recent years. As a precious remedial heritage of Chinese medicines, *TT* deserves to be further explored. However, no review of this research on the whole has been presented to date.

Therefore, in this present review, the phytochemistry and pharmacological effects on *TT*, have been comprehensively summarised to provide a solid base for further research. Such a systematic review on *TT* may be valuable for promoting its development into modern pharmaceuticals and improving its clinical uses.

## Methodology

This literature review of *Tadehagi triquetrum* was conducted from 1979 to October, 2021, on information from multiple popular electronic databases, including Science Direct, PubMed, Wiley, Google Scholar, ACS publications, SpringerLink, and China National Knowledge Internet (CNKI). Plant taxonomy was verified by ‘iPlant’ database (http://www.iPlant.cn). Additional information was also collected from Chinese herbal medicine books (in our school library), PhD and MSc theses (downloaded from CNKI), read manually and analysed in groups. Key words used in the literature search were: *Tadehagi triquetrum*, phytochemistry, pharmacology, pharmaceutical activities, chemical constituents, and other related search terms. Relevant reports and articles that appeared in some news media or newspapers, as well as literature that was not published in formal professional magazines and periodicals, have been excluded.

### Phytochemistry

**Figure 1.**
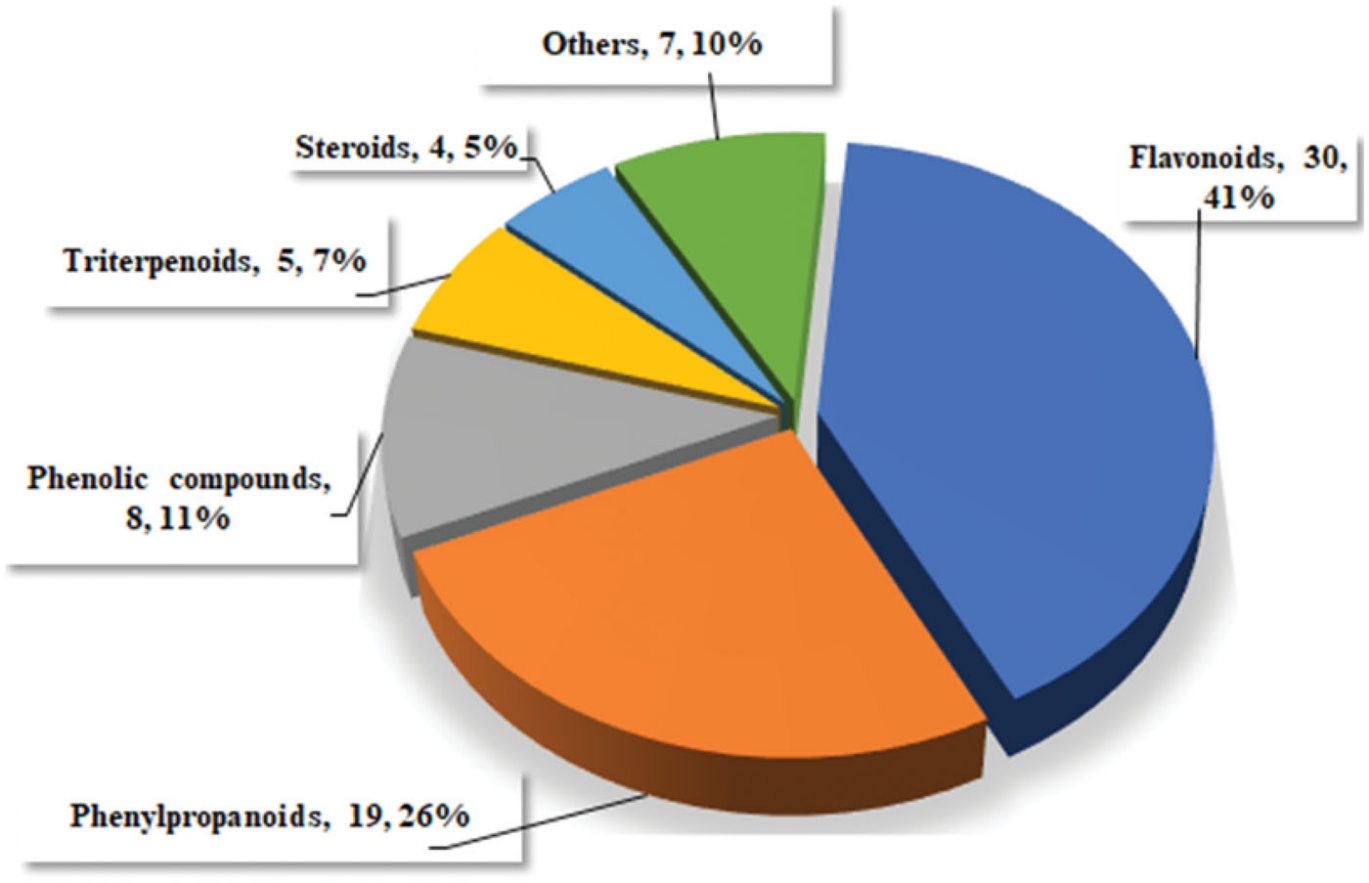
Structure classes from *Tadehagi triquetrum*.

**Figure 2.**
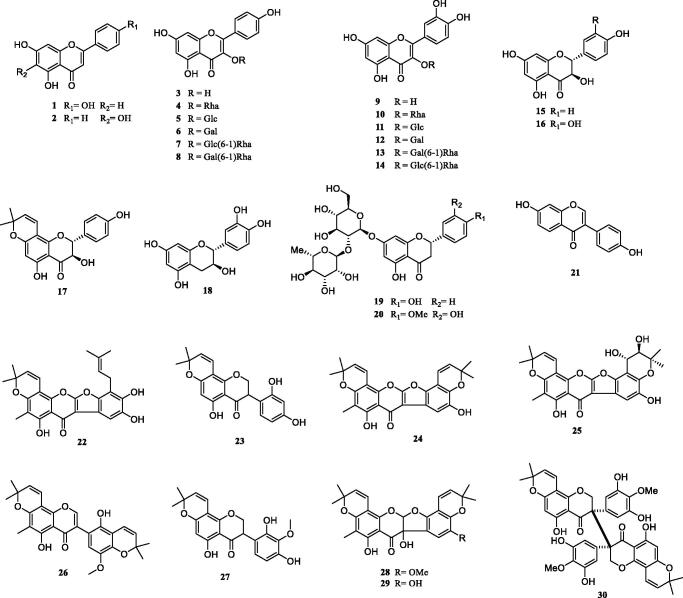
The structures of flavonoids from *Tadehagi triquetrum*.

**Figure 3.**
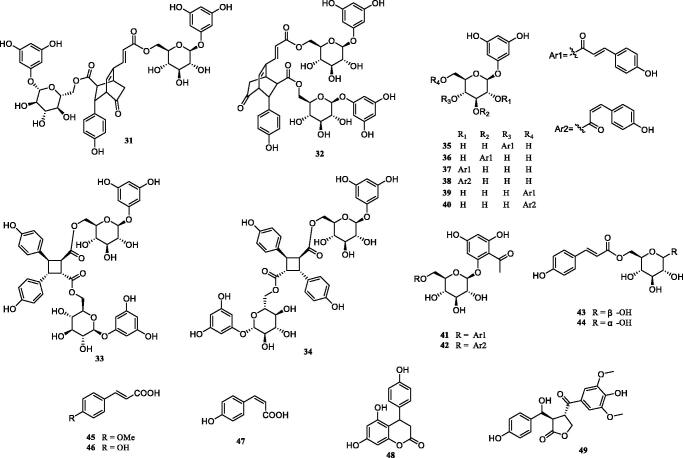
The structures of phenylpropanoids from *Tadehagi triquetrum*.

**Figure 4.**
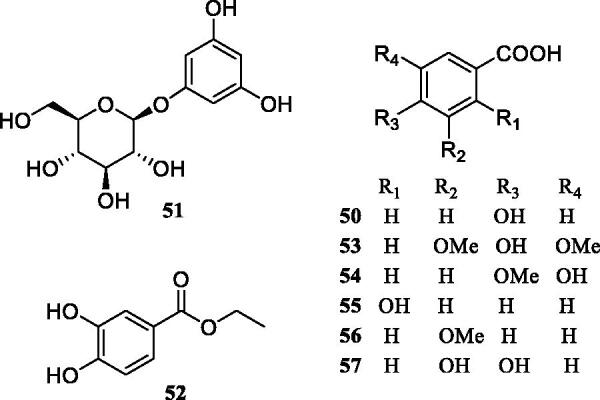
The structures of phenolic compounds from *Tadehagi triquetrum*.

**Figure 5.**
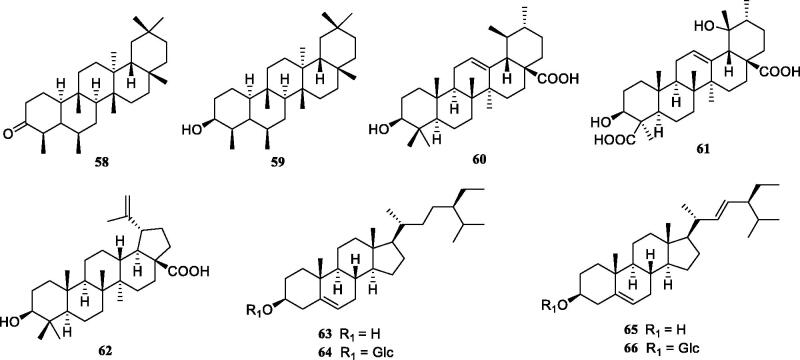
The structures of triterpenoids and steroids from *Tadehagi triquetrum*.

**Figure 6.**
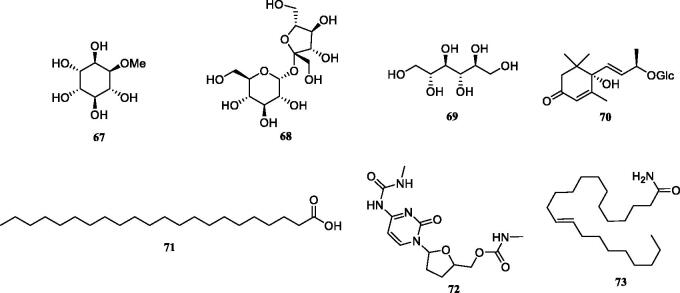
The structures of other compounds from *Tadehagi triquetrum*.

Previous investigation into the chemical constituents of extracts of *TT* have led to the isolation of flavonoids (**1**-**30**), phenylpropanoids (**31**-**49**), phenolic compounds (**50**-**57**), triterpenoids (**58**-**62**), steroids (**63**-**66**) and other miscellaneous compounds (**67**-**73**) (Figure 1). The chemical structures of these compounds are shown in Figures 1-6, and their names, skeleton types, molecular formula and related information are compiled in Table 2.

**Table 2.** Secondary metabolites isolated from *T. triquetrum*.

Classes	No.	Compound	Molecular formular	Part of plant	Source	Refence
**Flavonoids**	**1**	4′,5,7-Trihydroxyl-flavone	C_15_H_10_O_5_	aerial part	Guangxi, China	Wen et al. 1999
	**2**	Baicalein	C_15_H_10_O_5_	roots	–	Unni Jayaram et al. 2020
	**3**	Kaempferol	C_15_H_10_O_6_	whole plant; aerial part	Guangxi; Xishuangbanna; Hainan; China	Xiang et al. 2005; Zhou et al. 2013; Hu et al. 2017
	**4**	Kaempferol-3-*O*-*α*-L-rhamnoside	C_22_H_22_O_10_	aerial part	Guangxi; Hainan; China	Zhou et al. 2013; Hu et al. 2017
	**5**	Kaempferol-3-*O*-*β*-D-glucopyranoside	C_21_H_20_O_11_	aerial part; whole plant	Guangxi; Xishuangbanna; Hainan;China	Wen et al. 2000; Xiang et al. 2005; Zhou et al. 2013; Hu et al. 2017
	**6**	Kaempferol-3-*O*-*β*-D-galactopyranoside	C_21_H_20_O_11_	aerial part	Guangxi; Hainan; China	Wen et al. 2000; Hu et al. 2017
	**7**	Kaempferol-3-*O*-*β*-D-rutinoside	C_27_H_30_O_15_	aerial part	Guangxi, China	Wen et al. 1999; Zhou et al. 2013
	**8**	3-*O*-*β*-D-galactopyranosyl (6-1)- *α*-L-rhamnosyl-kaempferol	C_27_H_30_O_15_	aerial part	Guangxi, China	Wen et al. 2000; Zhou et al. 2013
	**9**	Quercetin	C_15_H_10_O_7_	aerial part	Hainan, China	Hu et al. 2017
	**10**	Quercetin-3-*O*-*α*-L-rhamnoside	C_21_H_20_O_11_	aerial part	Guangxi, China	Zhou et al. 2013
	**11**	Quercetin-3-*O*-*β*-D-glucopyranoside	C_21_H_20_O_12_	aerial part	Guangxi; Hainan; China	Wen et al. 1999; Zhou et al. 2013; Hu et al. 2017
	**12**	Quercetin-3-*O*-*β*-D-galactopyranoside	C_21_H_20_O_12_	whole plant	Hainan, China	Wu et al. 2014
	**13**	3-*O*-*β*-D-Galactopyranosyl (6-1)- *α*-L-rhamnosyl-quercetin	C_27_H_30_O_16_	aerial part	Guangxi, China	Wen et al. 1999; Zhou et al. 2013
	**14**	Rutin	C_27_H_30_O_16_	aerial part	Guangxi, China	Wen et al. 2000; Zhou et al. 2013
	**15**	Aromadendrin	C_15_H_12_O_6_	whole plant	Xishuang-banna, China	Xiang et al. 2005
	**16**	Taxifolin	C_15_H_12_O_7_	whole plant	Hainan, China	Jin et al. 2015
	**17**	Yukovanol	C_20_H_18_O_6_	whole plant	Xishuang-banna, China	Xiang et al. 2005
	**18**	(+)-Catechin	C_15_H_14_O_6_	aerial part	Guangxi, China	Wen et al. 2000; Zhou et al. 2013
	**19**	Naringin	C_27_H_32_O_14_	roots	–	Unni Jayaram et al. 2020
	**20**	Neohesperidin	C_28_H_34_O_15_	roots	–	Unni Jayaram et al. 2020
	**21**	4′, 7-Dihydroxyl-isoflavone	C_15_H_10_O_4_	aerial part	Guangxi, China	Wen et al. 1999
	**22**	Triquetrumone A	C_26_H_24_O_7_	whole plant	Xishuangbanna, China	Xiang et al. 2005
	**23**	Cyclokievitone	C_20_H_18_O_6_	whole plant	Xishuangbanna, China	Xiang et al. 2005
	**24**	Triquetrumone B	C_26_H_22_O_7_	whole plant	Xishuangbanna, China	Xiang et al. 2005
	**25**	Triquetrumone C	C_26_H_24_O_9_	whole plant	Xishuangbanna, China	Xiang et al. 2005
	**26**	Triquetrumone E	C_27_H_26_O_7_	whole plant	Xishuangbanna, China	Zhang et al. 2010
	**27**	Triquetrumone F	C_21_H_22_O_7_	whole plant	Xishuangbanna, China	Zhang et al. 2010
	**28**	Triquetrumone G	C_27_H_26_O_8_	whole plant	Xishuangbanna, China	Zhang et al. 2010
	**29**	Triquetrumone H	C_26_H_24_O_8_	whole plant	Xishuangbanna, China	Zhang et al. 2010
	**30**	(R)-triquetrumone D	C_42_H_38_O_14_	whole plant	Xishuangbanna, China	Xiang et al. 2005
**Phenylpropanoids**	**31**	Tadehaginoside A	C_42_H_44_O_20_	aerial part	Hainan, China	Zhang et al. 2016
	**32**	Tadehaginoside B	C_42_H_44_O_20_	aerial part	Hainan, China	Zhang et al. 2016
	**33**	Tadehaginoside C	C_42_H_44_O_20_	aerial part	Hainan, China	Zhang et al. 2016
	**34**	Tadehaginoside D	C_42_H_44_O_20_	aerial part	Hainan, China	Zhang et al. 2016
	**35**	Tadehaginoside E	C_21_H_22_O_10_	aerial part	Hainan, China	Zhang et al. 2016
	**36**	Tadehaginoside F	C_21_H_22_O_10_	aerial part	Hainan, China	Zhang et al. 2016
	**37**	Tadehaginoside G	C_21_H_22_O_10_	aerial part	Hainan, China	Zhang et al. 2016
	**38**	Tadehaginoside H	C_21_H_22_O_10_	aerial part	Hainan, China	Zhang et al. 2016
	**39**	Tadehaginoside	C_21_H_22_O_10_	aerial part	Hainan, Guangxi, China	Wen et al. 2000; Zhang et al. 2016; Hu et al. 2017
	**40**	6′-*O*-*cis*-*p*-Coumaroyl-3,5-Dihydroxyphenyl *β*-D-Glucopyranoside	C_21_H_22_O_10_	aerial part; whole plant	Hainan, China	Hu et al. 2017; Wu et al. 2014
	**41**	Tadehaginoside I	C_23_H_24_O_11_	aerial part	Hainan, China	Zhang et al. 2016
	**42**	Tadehaginoside J	C_23_H_24_O_11_	aerial part	Hainan, China	Zhang et al. 2016
	**43**	6-*O*-(*E*)-*p*-Hydroxy-cinnamoyl-*β*-Glucose	C_15_H_18_O_8_	whole plant	Hainan, China	Wu et al. 2014
	**44**	6-*O*-(*E*)-*p*-Hydroxy-cinnamoyl-*α*-Glucose	C_15_H_18_O_8_	whole plant	Hainan, China	Wu et al. 2014
	**45**	*tran-p*-Methoxycinnamic acid	C_10_H_10_O_3_	aerial part	Guangxi, China	Wen et al. 1999
	**46**	*tran-p*-Hydroxycinnamic acid	C_9_H_8_O_3_	aerial part; whole plant	Guangxi ; Xishuangbanna; Hainan; China	Xiang et al. 2005; Zhou et al. 2013; Jin et al. 2015
	**47**	*cis-p*-Methoxycinnamic acid	C_9_H_8_O_3_	whole plant	Hainan, China	Jin et al. 2015
	**48**	3,4-Dihydro-4-(4-hydroxyphenyl)-5, 7-Dihydroxycoumarin	C_15_H_12_O_5_	whole plant	Hainan, China	Jin et al. 2015; Wu et al. 2014
	**49**	Tadehaginosin	C_20_H_20_O_8_	aerial part	Hainan, China	Wu et al. 2014
**Phenolic compounds**	**50**	*p*-Hydroxybenzoic acid	C_7_H_6_O_3_	aerial part; whole plant	Guangxi; Hainan; China	Wen et al. 1999; Jin et al. 2015
	**51**	Phloroglucinol-*O*-*β*-D-Glucopyranoside	C_12_H_16_O_8_	aerial part	Guangxi, China	Zhou et al. 2013
	**52**	Ethyl 3,4-Dihydroxybenzoate	C_9_H_10_O_4_	whole plant	Hainan, China	Jin et al. 2015
	**53**	Syringic acid	C_9_H_10_O_5_	whole plant	Hainan, China	Jin et al. 2015
	**54**	3-Hydroxy-4-Methoxybenzoic acid	C_8_H_8_O_4_	whole plant	Hainan, China	Jin et al. 2015
	**55**	Salicylic acid	C_7_H_6_O_3_	leaves	–	Lv et al. 1995
	**56**	Vanillic acid	C_8_H_8_O_4_	aerial part	Hainan, China	Hu et al. 2017
	**57**	Protocatechuic acid	C_7_H_6_O_4_	leaves; aerial part	Hainan, China	Lv et al. 1995; Hu et al. 2017
**Triterpenoids**	**58**	Friedelin	C_30_H_50_O	stems	–	Yang et al. 1989
	**59**	Epi-fridelinol	C_30_H_52_O	stems	–	Yang et al. 1989
	**60**	Ursolic acid	C_30_H_48_O_3_	aerial part; whole plant	Guangxi; Xishuangbanna China	Wen et al. 2000; Xiang et al. 2005
	**61**	Ilexgenin A	C_30_H_46_O_6_	aerial part	Guangxi, China	Wen et al. 2000
	**62**	Betulinic acid	C_30_H_48_O_3_	whole plant	Xishuangbanna, China	Xiang et al. 2005
**Steroids**	**63**	*β*-sitosterol	C_29_H_50_O	whole plant	Xishuangbanna, China	Xiang et al. 2005
	**64**	Daucosterol	C_35_H_60_O_6_	aerial part; whole plant	Guangxi; Xishuangbanna China	Wen et al. 1999; Xiang et al. 2005
	**65**	Stigmasterol	C_29_H_48_O	stems	–	Yang et al. 1989;
	**66**	Stigmasta-5,22-Dien-3-*O*-*β*-D-Glucopyranoside	C_35_H_58_O_6_	whole plant	Xishuangbanna, China	Xiang et al. 2005
**Miscellaneous compounds**	**67**	2-*O*-Methyl-L-Chiro-Inositol	C_7_H_14_O_6_	whole plant	Xishuangbanna, China	Xiang et al. 2005
	**68**	Saccharose	C_12_H_22_O_11_	whole plant	Xishuangbanna, China	Xiang et al. 2005
	**69**	Galactitol	C_6_H_14_O_6_	whole plant	Xishuangbanna, China	Xiang et al. 2005
	**70**	Roseoside	C_19_H_30_O_8_	aerial part	Guangxi, China	Zhou et al. 2013
	**71**	Docosanoic acid	C_22_H_44_O_2_	whole plant	Xishuangbanna, China	Xiang et al. 2005
	**72**	(5-(4-[(Methylcarbamoyl) Amino]-2-Oxopyrimidin-1(2H)-yl) Tetrahydrofuran-2-yl) Methyl methyl carbamate	C_13_H_19_N_5_O_5_	roots	Uttar Pradesh, India	Srikanth Jupudi et al. 2019
	**73**	13-Docosenamide	C_22_H_43_NO	roots	Uttar Pradesh, India	Srikanth Jupudi et al. 2019

### Flavonoids

Flavonoids are one of the most common secondary metabolites in nature and have been found to possess numerous biological activities, which are known as possessing the C6-C3-C6 skeleton. They can be divided into many structure types based on the differences in the central C ring. Among all the constituents reported in this plant, flavonoids are the most abundant and have exhibited anthelminthic bioactivity. Up to now, 30 flavonoids (**1**-**30**) (Figure 2) have been isolated and identified from this plant (Wen et al. 1999, 2000; Xiang et al. 2005; Zhang, Cheng, et al. 2011; Zhou et al. 2013; Wu et al. 2014; Jin et al. 2015; Hu et al. 2017; Unni Jayaram et al. 2020) including flavones (**1**-**2**), flavonols and their glycosides (**1**-**14**), flavanonols (**15**-**17**), flavanone (**19**,**20**), one flavan-3-ol (**18**), isoflavonoids (**21**-**30**). Some of them exist as flavonoid glycosides and the aglycones in all of these glycosides are derived from flavonols and flavanones, which are mainly formed by the glycosylation of glucose, rhamnose and galactose. The C-5 and C-7 of *TT* flavonoids are oxidised in the form of hydroxylation or glycosylation. Investigation on the ethanol extract of the whole plant using bioactivity-guided isolation method led to the discovery of anthelminthic activity of three isoflavonoids (**22**, **24**, **25**) (Xiang et al. 2005). Isoflavonoids are very narrow in distribution and are very rare in other families of plants, except the Fabaceae. They are usually a major group of phytoestrogens possessing a wide variety of biological activities. Fabaceae plants are famous for their isoflavonoids, which are almost restricted to them. The structural feature of these isoflavonoids from *TT* is that they almost have one or two isoprenoid units (Zhang et al. 2011). Compound **30** is considered as the first bi-isoflavanone possessing a C-3/C-3′′′ linkage, which is an extremely rare carbon-carbon linked natural flavonoid dimer.

### Phenylpropanoids

Phenylpropanoids are also major compounds and active constituents in *TT*. Nineteen phenylpropanoids (**31**-**49**) have been isolated and identified from *TT* (Xiang et al. 2005; Zhou et al. 2013; Jin et al. 2015; Wu et al. 2015; Hu et al. 2017), most of which exist in the glycoside form and have been called tadehaginoside derivatives (Figure 3). These tadehaginoside derivatives generally contain three parts: a phloroglucinol, a glucosyl and a *p*-hydroxycinnamoyl. Such compounds were first reported in 2000 (Wen et al. 2000). These four compounds (**31**-**34**) from *TT*′s aerial part are rare dimeric tadehaginoside derivatives, which have a unusual bicyclo [2.2.2] octene skeleton or cyclobutene ring (Zhang et al. 2016). The plausible biogenetic pathway of their dimerisation could be through a [2 + 2] cyclisation reaction. In screening for hypoglycaemic activity, these above dimers could stimulate glucose uptake in C2C12 mouse skeletal muscle myotubes. It is worth noting that phloroglucinol and its derivatives have been widely reported due to their structural novelty and the diversity of bioactivities. Clinically, it is used as non-atropinic antispasmodic agent, which can directly act on smooth muscle (Li et al. 2011). The absolute configuration of tadehaginosin (**49**) was determined by NOESY correlations and optical rotation values compared with the reported literature (Li et al. 2011).

### Phenolic compounds

Up to now (Figure 4), eight phenolic compounds (**50**-**57**) have been identified from *TT* (Lv et al. 1995; Jin et al. 2015; Zhang et al. 2016; Hu et al. 2017). Many of these constituents possess multiple hydroxyl groups and are called polyphenols, most of which are weakly acidic and are modified by methoxylation and glycosylation or oxidisation. Due to the presence of hydroxyl groups, these phenolic compounds are considered to be major contributors to the antioxidant activity of *TT*.

### Triterpenoids

Five triterpenoids (Figure 5) have been isolated from *TT*, which are pentacyclic triterpenes including three skeletons: friedelane, ursane, and lupane type. Friedelane-type triterpenoids (**58**-**59**) from *TT*′s stems part are lipid-soluble component and they can be crystallised in solvents with low polarity such as petroleum ether mixed with chloroform (Yang et al. 1989). The latter three compounds (**60**-**62**) have increased their hydrophilicity due to the presence of carboxyl groups which could be converted from methyl oxidation at C-4 or C-28 (Wen et al. 2000; Xiang et al. 2005).

### Steroids

So far, four steroids (**63**-**66**) have been isolated from *TT* (Figure 5), in the free or glycoside forms (Yang et al. 1989; Xiang et al. 2005).

### Miscellaneous compounds

In addition, several miscellaneous compounds (Figure 6) have been described from *TT* (Zhou et al 2013; Xiang et al. 2005; Lv et al. 1995; Hu et al. 2017; Srikanth Jupudi et al. 2019), including three polyols (**67**-**69**), one roseoside (**70**), one fatty acid (**71**) and two alkaloids (**72**-**73**).

### Pharmacology

Modern pharmacological studies and plentiful clinical research literature have indicated that various extracts and purified compounds from *TT* had extensive range of biological activities.

### Anti-inflammatory effects

The animal models of inflammation were performed by carrageenan-stimulated rats. An ethanol extract from the leaves of *TT* was evaluated with measuring the paw volumes at 1 h intervals for 3 h and comparing with control groups. The results suggested it at all the doses (100, 200, and 300 mg/kg) could significantly decrease paw volume compared to control (*p* < 0.05) and the maximum inhibition of paw edoema was found at 60 min in the 300 mg/kg dose group, which were comparable with standard drug diclofenac sodium. In addition, over-production of nitric oxide (NO) indicates tissue damages. It from 25 to 75 μg/mL exhibited moderate inhibitory of NO in a dose dependent manner. Besides, the carrageenan administration process also could result in increasing in cyclic adenosine monophosphate (AMP)-phosphodiesterase. In a phosphodiesterase activity test, It showed better inhibitory effect against cyclic AMP phosphodiesterase than the positive control, mefloquine. (Kalyani et al. 2011).

### Hepatoprotective effects

Hepatopathy is featured by liver dysfunction and subsequent complications through inflammatory and oxidative stress occurred in hepatocytes. In China, traditional Chinese medicines (TCMs) including a large number of ethnologic herbs, play an important role in the treatment of various diseases. *TT*, as an old folk remedy, has been used very popularly by the Zhuang, Wa and Dai ethnic minority people and its long period of clinical application suggests *TT* has significant therapeutic effects for hepatopathy (Peng et al. 2008). Wa people in the folk often use the herbal prescription with *TT* as the principal medicine or monarch drug in TCM theory, to cure hepatitis and jaundice, and the *TT* decoction with stewed pork has been applied to treat hepatitis without jaundice. *In vitro* experiments on mice, the hepatoprotective effect of 70% ethanol extract of *TT* was investigated from the aspects of reducing transaminase, resisting oxygen free radicals, inhibiting lipid peroxidation and cytochrome P450 (Tang et al. 2016).

Carbon tetrachloride (CCl_4_) is considered as a hepatotoxic revulsant by generating reactive species, such as trichloromethyl radical (CCl_3_^−^), to destroy hepatocellular cells and undermine the liver functions. The hepatoprotective effect of tadehaginoside (**38**) isolated from *TT*′s aerial part was investigated using the hepatic injury model of CCl_4_-lesioned rats. The findings showed that it dose-dependently suppressed the cell proliferation of HepG2 cells *in vitro*, and it significantly lowered the serum concentrations of alanine aminotransferase (ALT), aspartate aminotransferase (AST), immunoglobulin E (IgE), and leukotriene (LT). The pathological examination suggested the hepatocellular damage was effectively mitigated by its treatment. Moreover, cytochrome c oxidase (COX) mRNA expression in hepatocytes was upregulated (Tang et al. 2020).

With the treatment of **38**, the levels of nuclear factor E2-related factor 2 (-Nrf2) and Kelch-like ECH-associated protein 1 (Keapl) were progressively increased, and the downstream enzymes consisting of γ-glutamylcysteine synthetase (γ-GCS), glutathione (GSH), and catalase (CAT), were activated and their contents in hepatocytes were gradually elevated, which may be involved with the downregulation of tumour necrosis factor alpha (TNF-α) and nuclear factor-kappa B (NF-κB)-expressed protein (Tang et al. 2014). In addition, **38** showed inhibitory effect on hepatic fibrosis induced by CCl_4_ in mice and significant downregulation the activities of caspase-3 and caspase-8. These findings demonstrated that **38** from *TT* could be used to develop an effective hepatoprotective drug (Tang et al. 2017a).

### Anti-hepatitis B virus effects

The inhibitory effect against hepatitis B surface antigen (HBsAg) of *TT* was first reported in 1986, which has no dose dependence (Xie et al. 1986). Previously, there have also been reports of a Chinese herbal decoction consisting of *Artemisia capillaris* Thunb., *TT*, *Sargentodoxa cuneata* R. and *Elephantopus scaber* L., to cure acute infectious hepatitis. Modern research found compound **38** can suppress the secretion of HBsAg, HBeAg and HBV-DNA from HepG2.215 cells in a dose-dependent manner, and the treated group with 40 μg/mL exhibited highest inhibitory of 83.2% against HBV-DNA, which was better than that of the nucleoside antiviral drug lamivudine (Tang et al. 2017b). Moreover, it can significantly increase the levels of intracellular signal transduction factors STAT1 and STAT2 mRNA.

Gannin formula is used to treat hepatic ascites clinically and has the characteristics of TCM and Zhuang medicine, including eight herbal medicines. Based on TCM theory, *Plumbago zeylanica* L. and *Carapax trionycis* were the monarch drugs, *Astragalus propinquus* Schischkin, *Atractylodes macrocephala* Koidz, *Paeonia lactiflora* Pall. and *Angelica sinensis* (Oliv.) Diels were the minister drugs, *TT* and *Lycopus lucidus* Turcz. Ex Benth. were adjuvant drugs (Yang et al. 2017). Cirrhosis ascites is one of the complications after hepatitis B develops into cirrhosis. Ganning formula could alleviate cirrhosis ascites and the remission rate of ascites reached 93.75% in the treatment group and 44.44% in the control group, with a significant different between the two group (*p* < 0.01) (Qin et al. 2016).

### Antidiabetic effects

In the type 2 diabetic mouse model established by intraperitoneal injection of streptozotocin (STZ, 150 mg/kg), the ethyl acetate fraction, *n*-butanol fraction and 60% ethanol fraction could significantly reduce the level of fasting blood glucose. Of these, 60% ethanol extract of *TT*′s stems and leaves exhibited the most significant hypoglycaemic effect and its hypoglycaemic rate reached 23.61%. Chloroform fraction and petroleum ether fraction had no activity on diabetic mice (Li et al. 2012). The latest study found the *n*-butanol fraction from *TT* shown the α-glucosidase inhibitory on yeast and small intestine in mice (He et al. 2020). Therefore, the effective constituent of antidiabetics from *TT* maybe mainly exist in the n-butanol fraction, and a detailed investigation is necessary to identify the chemical basis which is responsible for its antidiabetic activity.

The author utilised *n*-butanol to extract the 70% ethanol extract of *TT*′s stems and leaves to prepare the polyphenol-rich extract (PRE). It could lower hyperinsulinemia, improve oral glucose tolerance, and reduce hyperlipidaemia and liver fat content (*p* < 0.05). PRE treatment could increase the content of liver glycogen, and the activity of hepatic glucokinase and pyruvate kinase (*p* < 0.05). The eight compounds were isolated from PRE based on the bioactivity-guided isolation method. Six polyphenols (compounds **7**, **8**, **14**, **31**, **34**, **39**), particularly rutin (**14**), increased glucose consumption by hepatocytes, suggesting that polyphenols accounted for the antidiabetic effect of PRE (Lin et al. 2020).

Hyperglycaemia and insulin resistance (IR) are main characteristics of type 2 diabetic (T2DM). The activity of tadehaginosin (**49**) on consumption of glucose was evaluated on HepG2 cells. In the NBD-glucose (2-NBDG) uptake assay, its activity (10 mM) was comparable to insulin (0.1 mM), indicating a potent activity in stimulating glucose uptake by myotubes. Very interestingly, tadehaginosin (**49**) showed stronger activity than compound **48**, since they belonged to two structurally related phenylpropanoids, one lignan and one cumarin. The results suggested tadehaginosin could be a potential therapeutic strategy for stimulating glucose consumption and be meaningful in treatment of diabetes (Wu et al. 2015).

In another study, compound **34** exhibited the hypoglycaemic activity, with an efficacy comparable to that of 100 nM insulin in the model of C2C12 mouse skeletal muscle myotubes (Zhang et al. 2016). Molecular docking results demonstrated that **34** could bind tightly to peroxisome proliferator-activated receptor γ (PPARγ), a key regulator of glucose homeostasis. A series experiments of molecular biology showed that **34** significantly enhanced the transcriptional activity of PPARγ and increased the level of glucose transporter-4 (GLUT-4) protein whose expression was regulated by PPARγ. Therefore, the above results indicate compound **34** is regarded as a most prominent compound for the treatment of diabetes.

### Anti-gout effects

Zhuang medicines have been proved to effectively relieve clinical symptoms and improve immune function. ‘Jinqian Hulu’ decoction, as a traditional Zhuang prescription containing *TT*, has been used clinically in treatment of gout for decades and has achieved good clinical efficacy. Pharmacological studies *in vitro* have shown that the decoction could reduce the degree of toe swelling in mice with sodium urate injection and inhibit the synthesis of TNF-α and IL-1. Furthermore, it can significantly decrease the level of uric acid in hyperuricaemia mice of yeast intragastric administration, and suppress the activities of xanthine oxidase (XOD) and adenosine deaminase (ADA) which are key enzymes for purine metabolism to produce uric acid and a key link in the regulation of uric acid production (Tan et al. 2016). It is preliminarily shown that ‘Jinqian Hulu’ decoction has the effect of treating acute gouty arthritis, providing a theoretical basis for its further clinical use.

‘Hulucha Chubi’ decoction is another empirical formula based on traditional theory of Zhuang medicine, which contains four herbs with *TT* as the primary medicine. The clinical trial showed that this decoction could improve the symptoms and signs of acute gouty arthritis, which is an acute aseptic inflammatory response whose characteristic is intense pain caused by urate deposition. The results indicated its effect of reducing uric acid was significantly better than that of the control drug Diclofenac sodium Dual Release Eneric-coated Capsules (Temmler Ireland Ltd.) (Zheng and Huang 2017). The results confirmed its effectiveness and the validity of traditional medicine theory, and further provided the basis for its rational clinical application for gout arthritis.

### Anti-atherosclerotic effects

Scientific investigations indicated that compounds **35** and **41** significantly reduced oxLDL-induced lipid accumulation and exhibited comparable effects to that of the positive control simvastatin. Compounds **35**, **39** and **41** (10 μmol/L) prevented foam cells formation in RAW264.7 cells. And above three compounds could up-regulate of cholesterol efflux-related genes such as ABCA1 and ABCG1, and inhibit the expression of CD36 and SR-1, which are cholesterol influx-related genes. The structure-activity relationship study showed cinnamyl is the most pivotal group of these compounds (Wang et al. 2017).

### Antihyperlipidemic effects

Tadehaginoside (**38**) can decrease oleic acid (OA)-elicited neutral lipid accumulation and intracellular contents of total cholesterol and triglyceride in the HepG2 cell model (Wu et al. 2014). Its efficacy was comparable to that of the positive control simvastatin and was not due to its cytotoxicity. The PCR experiment indicated tadehaginoside decreased the transcription of lipogenesis-related transcription factors sterol regulatory element-binding proteins (SREBPs) and peroxisome proliferator-activated receptors (PPARs) (Zhang et al. 2015).

### Wound healing activities

Through clinical trials, Xiao have been used for the treatment of 89 cases of burn wounds and achieved good results from 1980 to 1994, using ‘Jiu-Bai-Cha’ lotion contained *TT*, *Senecio scandens* Buch.-Ham. ex D. Don, *Sanguisorba officinalis* L., and *Bletilla striata* (Thumb.) Rchb. f. The clinical observation found that it can relieve the burning pain, promote the blister subside and swelling decrease, prevent re-contamination and promote wound repair. The wounds of first-degree burns cured by this lotion generally did not leave paralysis marks, but only slight changes in the skin’s pigment. The lotion can also be used as an oil agent, which has little irritation on the wound surface and is suitable for children burns (Xiao 1995).

In another study, the results showed that the ethanol extract obtained from the leaves of *TT* could increase collagen deposition and hydroxyproline content in the incision wound model of rat. And the experiment in the excision wound model indicated it can enhance epithelization and collagenation to significantly increase in the rate of wound contraction. Thus, *TT* could be regarded as a medicinal remedy for wound healing (Shirwaikar et al. 2003).

### Antiparasitic and anthelminthic effects

‘Quchong Shengxue’ pills containing 51.6% *TT*, were clinically used to treat ancylostomosis with severe anaemia. The results showed that the clinical recovery rate reached 94.29% (Luo 2000).

The expelling effect of 10% of *TT* against rabbit coccidian was investigated using the McMaster’s method, which suggested its effective dose to rabbit coccidia was 0.5-1.3 g/kg. Above the dose of 1.9 g/kg, it caused death in rabbits (Li et al. 2002b). *In vitro* test, cyclokievitone (**23**) had remarkable effects on inhibiting or killing coccidian eggs (Li et al. 2003a). The above findings implied its potential in veterinary medicines.

### Anti-allergic effects

Allergic asthma is a disease characterised by chronic bronchitis and hyperreactivity of trachea involving multiple inflammatory cells. The 50% acetone extract of *TT*′s stems and leaves exhibited the anti-allergic effect on the rat model of type I allergy. It decreased the contents of IgE, leukotriene and histamine in the serum at the dose of 100 mg/kg. Compared with the positive control group, the treatment group with a dose of 100 mg/kg significantly reduced the number of eosinophilia and the area of inflammation in lung tissue (Zhou et al. 2011).

The researchers found *TT* had strong agonistic activity against G protein-coupled receptor-35 (GPR35) with high pressure liquid chromatography and the label-free method. HPLC-TOF-MS was utilised to analyse the ingredients of its active fractions and identify twelve compounds (**1**, **3**, **7**, **11**, **13**, **14**, **16**, **26**, **27**, **51**, **60** and **64**) (Ding et al. 2017).

### Anti-fungal and anti-bacterial effects

*TT* was found to be active in the anti-bacterial test by inhibiting cyclic AMP phosphodiesterase, with the inhibition ratio of 72.58 ± 1.89%, compared to the positive control mefloquine with 45.20 ± 3.30% (Chit et al. 2001). Zhang et al. described that it could be resistant to bacterial toxin (Zhang et al. 2011). Clinically, the combination of herbs that contains 20% of *TT*, has been used to treat the candida vaginitis, which had a 76.7% cure rate (Zhang 1979).

Infantile pustules are a common skin infection caused by *Staphylococcus aureus*. The joint use of *TT* and mupiroxine could make the cure rate of disease reach 60% (Tian 2013).

### Anti-mollusk effects

The crude powder and the decoction of *TT* had a good effect on the expulsion of Lymnaeidae mainly containing *Radix swinhoei* and *Galba pervia*, and the powder exhibited better effect. The powder group with the concentration of 0.5% (g/L) could kill all these molluscs within 17 hours (Li, Li et al. 2003). Besides, the tested extract of *TT* with the concentration of 1.6% also showed the anti-mullusk activity.

Compounds **46**, **67** and **69** showed no activity against *Radix swinhoei*, which belonged to water-soluble compounds, but cyclokieviton (**23**) and kaempferol (**3**) with the concentration from 0.005-0.01% had the activity within 24 hours. Thus, it was suggested that they might be effective components of *TT* (Li et al. 2003b).

### Antioxidative effects

*TT* is rich in polyphenolic compounds which have been considered to possess antioxidant activities. Because of the availability and the presence of flavonoids and phenolic compounds, 95% ethanol extract of *TT*’s leaves was evaluated for its antioxidant activity *in vitro*. The extract could decrease significantly the concentration of DPPH-radical and H_2_O_2_, whose effect on scavenging of free radicals was close to the control ascorbic acid. This extract at the concentration of 50 μg/mL had the maximum H_2_O_2_ scavenging effect (Kalyani et al. 2011).

### Treatment of anorexia and dyspepsia

Infantile anorexia is a common disease in paediatrics, which usually occurs in infants under one year old. The patients mainly have symptoms such as anorexia and fatigue. ‘Xiaoer Kang’ granule is a kind of Chinese patent medicine including *TT* (Wang and Zhang 2010). The results from the clinical test showed that the total effective rate and significant efficiency of the treatment group were significantly higher than those of the control group, and the treatment group had a better effect in improving the accompanying symptoms (Huang and Zheng 2017).

### Genitourinary function

Gao and Chen used the ‘Gongying Hulu’ decoction to treat prostate hyperplasia with the syndrome type of moist-heat on bladder in TCM (Gao 2004; Chen 2008). Sun has reported that the total effective rate of treating prostate hyperplasia using the decoction have reached 93.4% (Sun 2003). Meanwhile, the decoction has been used to treat 98 cases with prostate hyperplasia in combination with ‘Buzhong Yiqi’ pill, and the rate of clinical cure was 32.7% (Wang 1995). Wang and Xu have treated the acute urinary retention caused by prostatic hyperplasia and the effective rate of treatment was 78.6% (Wang and Xu 1998). Professor Jin has used *TT* to treat various genitourinary diseases (Zhang et al. 2010).

### Insect extermination effect

To verify the traditional record of deworming, its insect extermination effect was investigated towards the larvaes of Tenthredinidae and Arctiidae using its different dosage forms (Li et al. 2004). Since it actually was inactive against these larvae, the reported folk statement of ‘removing and killing insects’ may be understood as ‘expelling’ its adults so that they cannot lay eggs in the host, and then it is impossible for them to develop larvae, and another explanation was that the target of expulsion was the parasites.

### Other applications

Deng often substitute *TT* for *Artemisia capillaris* Thunb., a commonly used traditional Chinese medicine with the effect of reducing dampness and removing jaundice (Jin et al. 2017). Li summarised *TT*’s clinical applications as the main herb, such as treating carbuncle, heat dysentery and malnutrition (Ge and Li 1994). In addition, it can be used as feed additive with the purpose of promoting digestion and gaining weight in a mouse model (Li et al. 2002a).

## Conclusions

As a famous traditional medicine with a long history, *TT* has many medicinal applications. It can be used as a single medicine or in combination with other TCMs in multiple ethnic groups to treat various ailments. Some medicinal uses are clearly recorded in traditional medicine classics and official ethnic literature, while others have been directly used in folk life through generations of oral transmission. Just because of its clinical efficacy and history, more and more researchers are interested in it. So, extensive phytochemistry and pharmacology investigations on *TT* have been carried out. Therefore, this review described and summarised the detailed chemical constituents and their biological activities of *TT*, which could provide the foundation for future research. To the best of our knowledge, 73 compounds are present in this plant and mainly include flavonoids, phenylpropanoids, phenolic compounds, triterpenoids and steroids. Among them, some compounds show good pharmacological activities, especially anti-atherosclerotic and antidiabetic activities. Studies indicated the phenylpropanoids in *TT* had the potential in treatment of diabetes disease, but their structure-activity relationship and the mechanism of action required deeper research. Some studies have found single compounds with novel skeletons, but their activities seemed to be unsatisfactory.

It is particularly worth mentioning that the results of modern pharmacological research do not seem to be as good as it is used in real life. This may involve multiple factors, such as the authenticity of *TT*, collection time, processing methods, extraction conditions. In particular, the extraction method of *TT* is decoction in folk clinical application, but in most reports, it is reflux extraction by ethanol. So, it is very necessary to go into the clinic and study its preparation process in advance. Besides, in many cases, its extracts performed better than single compounds, suggesting that these single pure constituents could not well explain the traditional medicinal activities of *TT*. This may indicate its mechanism of action is related to multiple components. Hence, it is necessary to develop a variety of pharmacological models and methods, such as network pharmacology strategies, to promote the research of multiple components and multiple targets of TCMs.

To be sure, many of pharmacological studies have been conducted with its extract or its formulations, and many of them are based on *in vivo* studies. But many of the early experiments were very superficial. A relatively large amount of research has focussed on its extracts and even powders, without really finding its effective ingredients. Therefore, the studies based on monomeric compounds are still lacking, especially in terms of activity screening, action mechanism and pharmacodynamics. Furthermore, more clinical trials should be conducted to verify its functions. On the whole, the research is not systematic and in-depth enough, specifically for one therapeutic effect. Of course, suitable pharmacological experimental models *in vitro* and *in vivo*, are also very vital and urgent.

In addition, studies on its composition analysis and quality control, such as component fingerprint, have not yet established clear and reliable strategies. In the future, further research is necessary to investigate phytochemical and pharmacological activities, including the deeper molecular mechanism, in order to discover active compounds from *TT*.
